# Rally on the Great M. V. at St. Louis

**Published:** 1909-09

**Authors:** 


### EDITORIAL DEPARTMENT.

### RALLY ON THE GREAT M. V. AT ST. LOUIS.

You may paint,—you may whitewash the quack if you will,
But the odor of quackery will linger there still.

Notwithstanding the copious calcimining bestowed upon "our peerless leader" at Atlantic City, and the presentation of a watch by certain Chauvins from Texas—his name will ever be redolent of water cure and liquid oxygen, and the dissatisfaction with him and his methods grows apace and, sooner or later, will culminate in a large desertion of the Octopus standard.

Notwithstanding the report of the dictator, so much applauded, shows such gains in membership in ten years, the attendance at Atlantic City, the favorite resort, was only 3200, as against 6800 at Chicago in 1908, before the exposure of the outrageous quackery practiced by the great First Lord was published. There is no other way to account for this falling off. The subject has become odious. Many self-respecting members stay away.

An opportunity now presents for all self-respecting members—men who revere the traditions and history of scientific medicine, who would have a grand association of truly ethical physicians, free from contamination by admixture of aliens—quacks—osteopaths—pathies and negroes—free from the machinations and domination of an apostate and reformed (?) quack and a clique of interested "rooters," claquers,—to build up such an association. The great Mississippi Valley Medical Association meets next month (October 12, 13 and 14) in St. Louis. Let there be a large and enthusiastic attendance from every Southern State. Let them transfer their allegiance from the *omnum gatherum*—ye Octopus—to this Association; withdraw their support from the great journal that feeds them on husks, and gives them a stone for a fish, and establish a journal of the M. V. which shall be pure, ethical, impartial and something more than a weekly record of the doings of the immaculate oligarchy. On this subject Dr. Robert Powell of Denver (in *Southern Practitioner)* says:

"We look to see this matter develop into a revolt from the American Medical Association. When that part of the membership that is dissatisfied with the present management sees that it is impossible to effect the necessary reforms within the organization, we will see many of the best element dropping out. The organization is strictly voluntary; no physician needs to be a member unless he chooses. If he feels that he can not stand for what is being done in his name by the misrepresentatives who are maintained in their places through his contributions, he does not have to remain in the society.

"Only a minority of the physicians of the United States have ever enrolled themselves in this body. The number has been greatly increased recently, through the efforts of paid organizers, like 'Pardoned McCormick,' who have gone about the country gathering in numbers of those who never before united themselves with this association and who are held but slightly in allegiance to it. Despite all such efforts, carried out with the most lavish expenditure, the association still comprises a minority of American physicians. Dr. Daniel's bold defiance of 'the powers that be' seems likely to precipitate the tempest which has been hovering over the machine. The formation of a Southern Medical Association may be predicted as a probable outcome of the general dissatisfaction to which Daniel has given expression.

"Many advantages would attend the formation of such an association. The problems presented to the practitioner along the Gulf differ so radically from those in the rest of the country that an association limited to this section would be more practically useful to its members. The social conditions here render the medical course, as to its length and the studies in the college curriculum, so far peculiar, that much of the time spent in the Northern school is wasted by the Southern student, who is put to excessive expense to learn that for which he will have no possible use. Meanwhile he finds on going into practice that most of the diseases he is to treat were unknown to his teachers under the aspect in which they present themselves to his eye."

